# Value-Based Outsourcing Is Associated with Improved Healthcare Outcomes in Low- and Intermediate-Complexity European Hospitals: A Cross-Sectional Study from Spain

**DOI:** 10.3390/healthcare14060731

**Published:** 2026-03-13

**Authors:** Jorge Short Apellaniz, Bernadette Pfang, Ángel Blanco Rubio, Adriana Pascual, Ignacio Maestre Mulas, Raquel Barba-Martín, Ángel Jiménez, Antonio Nuñez García, Juan Antonio Álvaro de la Parra, Marta del Olmo Rodríguez

**Affiliations:** 1 Quirónsalud Red 3H, 28223 Madrid, Spain; 2 Unidad de Innovación Clínica y Organizativa, Hospital Universitario Fundación Jiménez Díaz, 28040 Madrid, Spain; 3 Instituto de Investigación Sanitaria Fundación Jiménez Díaz (IIS-FJD), 28015 Madrid, Spain; 4 Information Technology and Systems Department, Quirónsalud Healthcare Network, 28010 Madrid, Spain; 5 Medical Direction, Hospital Universitario Infanta Elena, 28342 Madrid, Spain; 6 Medical Direction, Hospital Universitario General de Villalba, 28400 Madrid, Spain; 7 Medical Direction, Hospital Universitario Rey Juan Carlos, 28933 Madrid, Spain; 8 Internal Medicine Department, Hospital Universitario Infanta Elena, 28342 Madrid, Spain; 9 Internal Medicine Department, Hospital Universitario General de Villalba, 28400 Madrid, Spain; 10 Management, Quirónsalud Healthcare Network, 28010 Madrid, Spain

**Keywords:** value-based healthcare, outsourcing, patient outcomes, efficiency, healthcare management, hospital management, healthcare outcomes, length of hospital stay, hospital acquired infection, inpatient complications, patient satisfaction

## Abstract

**Background:** Value-based healthcare (VBHC) has emerged as a promising approach for improving quality while reducing costs. While evidence from tertiary hospitals suggests that outsourcing to VBHC networks can improve safety, efficiency, and satisfaction, less is known about its impact in low- and intermediate-complexity hospitals. The Madrid Regional Health System (RMHS), which stratifies hospitals by complexity, provides a unique opportunity to compare performance across management models. The objective of this study was to compare outcomes between low and intermediate-complexity hospitals outsourced to a VBHC network with those operating under public management and outsourced to traditional for-profit organizations. **Methods:** The researchers conducted a cross-sectional analysis using the RMHS 2024 annual audit report. Sixteen low- and intermediate-complexity hospitals were included: three outsourced to the VBHC network Quirónsalud and thirteen under public management and outsourced to a traditional private for-profit network. Monographic and long-term facilities were excluded. Variables included case mix complexity, quality and safety indicators (inpatient complications, hospital-acquired infections, low-risk cesarean sections), efficiency metrics (average and case-mix-adjusted length of stay), and patient satisfaction measures (survey scores and patient transfers under the Free Choice of Care Mandate). Continuous variables were compared using Mann–Whitney U tests and categorical variables with Chi-square tests. **Results:** Study hospitals managed more complex patients (median case-mix 1.06 vs. 0.88, *p* = 0.007). Despite this, no differences were found in complication rates. Hospital-acquired infections (3.47% vs. 5.46%, *p* < 0.001) and low-risk cesarean sections (16.1% vs. 19.3%, *p* < 0.001) were significantly lower in VBHC hospitals. Efficiency was improved, with shorter average length of stay (4.26 vs. 5.03 days, *p* = 0.031) and all study hospitals demonstrating lower-than-expected case-mix-adjusted stay, unlike several controls. Patient satisfaction was higher (0.91 vs. 0.87, *p* = 0.007), as were recommendation scores (0.96 vs. 0.92, *p* = 0.003). Patient transfers favored outsourced hospitals, with more patients choosing them and fewer leaving compared with controls. **Conclusions:** Low- and intermediate-complexity hospitals managed by a value-based network in Madrid achieved superior performance across safety, efficiency, and satisfaction indicators, despite treating more complex patients. These findings extend evidence for VBHC outsourcing beyond tertiary hospitals, highlighting potential for improved system-wide performance where universal coverage and hospital stratification ensure comparability. VBHC outsourcing may represent a viable strategy to enhance patient outcomes and optimize resource use in regional healthcare systems.

## 1. Introduction

Evidence suggests that value-based healthcare (VBHC) is a potentially effective strategy for delivering high-quality healthcare at the lowest possible cost for patients and healthcare systems [1,2,3,4], which is its basic principle [5]. According to VBHC theory, the goal of value delivery can be achieved through a combination of strategies which include measuring outcomes and costs for every patient, building an enabling information technology platform, organizing care around the patient through the implementation of integrated practice units (IPUs), moving towards bundled payments for care cycles, integrating care delivery systems across different facilities, and expanding geographic reach [6,7,8]. Other, more recently described elements of VBHC theory include designing and implementing systematic approaches for value-based quality improvement; integrating the concept of value in patient communication; investing in a culture of value delivery; and building learning platforms for healthcare professionals based on patient outcome data [9,10,11].

In recent years, the interplay between public and private healthcare in a value-based environment has become a question of interest for the scientific community and policymakers [12,13]. In fact, in the European setting, it seems that value-based healthcare may drive collaboration between different care providers to benefit both patients and carers, as well as the broad healthcare ecosystem [14,15]. A scoping review cited various examples of value-based healthcare with collaboration between private and public entities, for example, the Netherlands Heart Network, that are associated with improved patient outcomes [13,16]. In a recent work, the researchers reported how a tertiary public hospital in Madrid, Spain, outsourced to a value-based private healthcare network, outperformed other publicly managed hospitals on a range of performance indicators, including safety, efficiency, and patient satisfaction scores [17]. However, evidence is lacking regarding the effect of value-based outsourcing in other, lower-complexity hospital settings.

While tertiary or high-complexity hospitals are often associated with more complex patients and have higher technological status and higher capacity, hospitals with a lower complexity, such as regional or provincial hospitals, serve as a first port-of-call for patients living in local neighborhoods and play an important role in providing health services in the community, as well as identifying patients with a need for more specialized care [18,19]. Under the value-based approach, the coexistence of high- and low-/mid-complexity hospitals managed by the same network is an opportunity to reorganize care across geography, to optimize the use of resources while increasing access to high-quality care to patients across catchment areas [5]. At the same time, under the value-based perspective, the coexistence of multiple degrees of complexity in different locations is a stimulus for healthcare providers to develop new models of care delivery to improve patient and professional experience and health results while lowering heath expenditure [5]. Despite these potential advantages to value-based outsourcing, no direct comparison has been carried out between low and intermediate complexity public hospitals managed by a value-based network and those managed by the local health system. While several healthcare systems such as China and India have a clear stratification system that enables direct comparisons, most European countries do not clearly identify different tiers of hospital complexity, thus making direct comparisons difficult [19,20]. The Regional Madrid Health System from Madrid, Spain, is one of the few European health systems to stratify hospitals into low, intermediate and high complexity, making direct comparison possible in the European context. At the same time, Spanish law permits the outsourcing of public hospitals to private healthcare networks, with several hospitals of the Regional Madrid Health System having been outsourced to the value-based network Quirónsalud for over a decade.

This study includes annual performance indicators from the regional health system of Madrid, Spain, comparing low- and intermediate-complexity hospitals outsourced to a value-based private healthcare network with those under public management and outsourced to traditional private networks.

## 2. Materials and Methods

### 2.1. Study Design

This was a cross-sectional study including annual audit data from the Regional Madrid Healthcare System’s 2024 report for low- and intermediate-complexity hospitals. The researchers aimed to compare indicators of quality of care and patient safety, efficiency, and patient satisfaction between low- and intermediate-complexity hospitals outsourced to a private, value-based healthcare network, and those managed by the local health system.

### 2.2. Study Setting

There are 34 public hospitals in the Regional Madrid Healthcare System (RMHS), of which 6 are defined as “low complexity” and 12 as “intermediate complexity”. Definitions of hospital complexity used by the RMHS are presented in Table 1. Some studies have pointed to the RMHS as offering the highest quality care of Spain’s 17 regional health systems. Several of the RMHS public hospitals are outsourced to private healthcare organizations, of which one low complexity hospital (Hospital Universitario Infanta Elena) and two intermediate complexity hospitals (Hospital Universitario Rey Juan Carlos and Hospital Universitario General de Villalba) are outsourced to the value-based healthcare network Quirónsalud. It should be noted that Spanish healthcare offers universal care at zero out-of-pocket cost to Spanish citizens and residents, and this norm is applicable both to publicly and privately managed public hospitals. Since 2011, the Free Choice of Care Mandate enables individuals in the Madrid Region to seek care at the hospital and primary care center of their choice, without being restricted to a specific catchment area.

### 2.3. Sampling

This study included 16 low- and intermediate-complexity hospitals from the RMHS. The three hospitals outsourced to the Quirónsalud healthcare network were the study group, with 12 publicly managed hospitals and one hospital outsourced to another traditional model network comprising the control group. The researchers excluded monographic (pediatric and psychiatric) hospitals, as well as long-term facilities.

### 2.4. Variables

The researchers described a number of care episodes during the study period, stratified as inpatient stays, outpatient visits, surgical procedures, and emergency department visits. The researchers also described case-mix complexity for each of the included hospitals. Regarding quality of care and patient safety metrics, the researchers included the standardized two-year mortality rate, the percentage of medical and surgical inpatient complications, and rates of hospital-acquired infection, as well as the number of low-risk cesarean sections performed during the study period. Regarding efficiency metrics, the researchers included hospital length of stay and case-mix-adjusted inpatient length of stay. Finally, the researchers included patient satisfaction scores as a direct indicator of patient experience, as well as numbers of patients choosing to transfer to and away from the different hospitals as indirect indicators. Operational definitions of healthcare outcomes and other variables included in the study are presented in Table 2.

### 2.5. Data Sources/Measurement

All data were extracted manually from the 2024 RMHS annual hospital audit report (see Supplementary Materials for the supporting dataset) [21]. This report features data from different central databases including mandatory information from hospital discharge reports, waiting list information, and patient satisfaction survey scores. Annual rates of inpatient surgical and medical complications were calculated by the RMHS for each hospital by dividing the number of discharged patients with one or more coded complications by the total number of discharges in that year, multiplied by 100. Low-risk cesarean sections were defined as those taking place in women in the absence of the following conditions: abnormal presentation, preterm birth, stillbirth, and multiple pregnancies [22]. The prevalence of hospital-acquired infections was reported by each hospital through point-prevalence surveys, following standardized methodology from the European Point Prevalence Survey (EPPS) model [23]. In order to compare rates of hospital-acquired infections between groups, we multiplied point-prevalence infection rates by the total number of inpatient stays for the study period.

Efficiency indicators included in this study covered the average length of hospital stay and case-mix-adjusted average inpatient length of stay (CMAILS). For the average length of stay, outliers were excluded to prevent distortion of results. CMAILS was calculated for each hospital by dividing the mean length of stay (in days) by a reference standard (the estimated length of stay in days, adjusted by case mix and based on the RMHS overall mean).

Patient satisfaction indicators consisted of annual results from the Madrid Department of Health’s patient experience surveys, as well as data on patient transfers between low- and intermediate-complexity hospitals under the Free Choice of Care Mandate [24]. Survey results were published in annual audit reports and expressed as scores from 0 to 100, where 0 indicated the poorest possible experience and 100 the best. Surveys were conducted via telephone with patients’ consent, and responses were recorded anonymously. Results were stratified by hospital and grouped across four dimensions of care: surgical care, inpatient care, outpatient care, and urgent care. Additionally, the number of patients opting to transfer to or from each hospital was collected from annual audit records.

In order to avoid selection bias, the researchers included all low- and intermediate-complexity hospitals of the RMHS, excluding only monographic hospitals such as pediatric and psychiatric hospitals, as well as long-term facilities. The risk of reporting bias was mitigated through the inclusion of solely official audit data.

### 2.6. Statistical Methods

Continuous variables were expressed as median [interquartile range] and qualitative variables as frequencies. Comparison between groups was performed using a Mann–Whitney U test for continuous variables and a Chi-square test for qualitative variables. In order to compare differences in surgical waiting times and average length of stay, data were transformed by multiplying each average by the total number of surgical procedures and inpatient stays, respectively, before carrying out the Mann–Whitney U test. Likewise, to compare rates of surgical and medical complications and hospital-acquired infection, variables were transformed by multiplying frequencies by total number of inpatients for each hospital. The percentage of low-risk cesarean sections was multiplied by the total number of births per hospital as a proxy to allow for comparisons between groups. We performed separate analysis for the low- and intermediate-complexity groups, as well as an overall analysis comparing all hospitals in the study and control groups. A *p*-value of less than 0.05 was considered statistically significant.

Adjusted indicators (case-mix-adjusted inpatient length of stay) allowed for comparison with the RMHS’s standardized reference values, set at 1. The RMHS calculated a 95% confidence interval for each result using Byar’s approximation of the exact Poisson distribution. Results were extracted from annual audit data. Length of stay was classified as lower than average when the corresponding confidence interval was entirely below 1, whereas it was classified as higher than average when both bounds of the confidence interval exceeded 1.

Analyses were carried out using Python v.3.1.

### 2.7. Ethics

The study adhered to the principles set forth in the Declaration of Helsinki [25]. As it featured only aggregated data in the public administration domain, no informed consent was required. The study received a formal ethics waiver from the Fundación Jiménez Díaz Research Ethics board.

## 3. Results

During the study period (1 January 2024–31 December 2024), a total of 7,684,462 care episodes were recorded, of which the study hospitals accounted for 1,743,090 (22.68%). A detailed description of care episodes and indicators is depicted in Table 3.

Regarding quality of care and patient safety indicators, median case-mix complexity was significantly higher for the study hospitals (1.06 versus 0.88, CI 0.10–0.27, *p* = 0.007). Despite the higher complexity of study hospitals, no differences were observed between rates of inpatient medical and surgical complications (2.56% versus 2.58%, *p* = 0.991). Rates of infection were significantly lower for the study group (3.47% versus 5.46%, *p* < 0.001). Lower rates of low-risk cesarean sections were also observed (16.10% versus 19.32%, *p* < 0.001).

**Figure 1:**
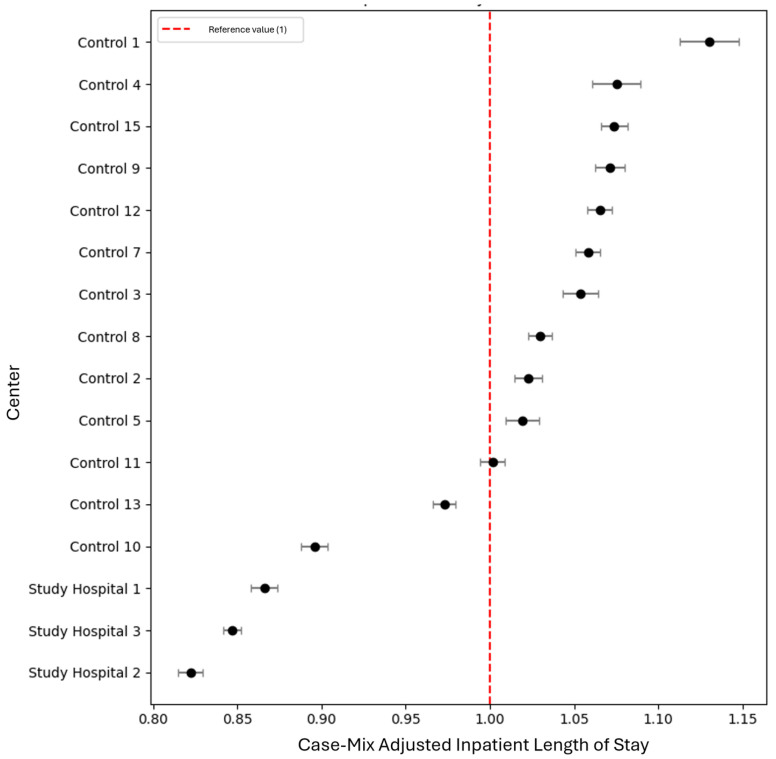
Forrest plot depicting case-mix-adjusted length of inpatient stay.

Regarding efficiency metrics, study hospitals demonstrated significantly lower average inpatient length of stay (4.26 versus 5.03, CI −0.95–−0.30, *p* = 0.031), while case-mix-adjusted length of stay was lower than expected for the three study hospitals, in contrast to control hospitals, of which 10 presented higher-than-expected case-mix-adjusted length of stay (Figure 1).

Upon analysis of patient satisfaction scores, the three study hospitals demonstrated significantly higher overall patient satisfaction scores (0.91 versus 0.87, CI 0.03–0.07, *p* = 0.007), as well as patient recommendation scores (0.96 versus 0.92, CI 0.03–0.05, *p* = 0.003). Finally, the median number of patients choosing to transfer to study hospitals was significantly greater than that of patients choosing to transfer to control hospitals (23,750 versus 3606, CI 8115–31,194, *p* = 0.014), while no differences were observed in numbers of patients choosing to transfer away from their catchment area hospital (3795 versus 13,640, *p* = 0.439).

## 4. Discussion

This study compared performance indicators from low- and intermediate-complexity hospitals managed under a value-based private healthcare network with those managed directly by the public health system in Madrid, Spain. Our findings suggest that hospitals within the value-based network achieved significantly better results across key domains of healthcare delivery, including quality of care, efficiency, and patient satisfaction, despite managing patients of greater case-mix complexity. These results provide initial evidence that value-based outsourcing may represent a viable strategy for improving care delivery in non-tertiary hospital settings.

One of the most notable findings was the significantly lower rate of hospital-acquired infections in study hospitals. Prevention of healthcare-associated infections is a well-established quality marker, and reductions in infection rates are directly associated with improved patient safety and lower costs of care [26,27,28]. Importantly, these improvements were achieved in hospitals serving more complex patients, suggesting that process standardization and organizational structures in value-based networks may contribute to stronger infection control and surveillance practices, including perioperative care, as demonstrated in earlier works [29,30]. Our findings contrast with studies from the United States failing to observe improved rates of healthcare-associated infection after widespread implementation of state-run value-based incentive programs [31,32]. These studies point to the importance of considering other determinants of health outcomes such as social and economic status and hypothesize that value-based incentive programs may be disadvantageous for “safety net” hospitals serving vulnerable populations, a point of view shared by other authors [33,34]. However, other studies have demonstrated that value-based models actually improve access to care and may reduce social disparities [35,36]. In our opinion, it is important to take into account the structure of different healthcare systems when seeking to evaluate the effects of value-based initiatives on clinical outcomes. Our study is set in the Spanish healthcare system which—contrary to the United States—offers universal access to healthcare at zero out-of-pocket cost for patients. Also, the Regional Healthcare System of Madrid operates under a free-choice policy, ensuring that residents can access the hospital of their choice with no restrictions [37]. In this context, it is probable that the effects of value-based healthcare delivery are more evident, due to the reduced risk of “cherry picking” in the context of universal access to care and free choice of healthcare provider.

As mentioned, a potential risk of “pay-for-performance” schemes and, indeed, outsourcing in general, is to prioritize more “profitable” patients to nominally improve outcomes for an economic benefit [33,34,38,39]. However, our results indicate that when outsourcing to a value-based network takes place in a healthcare ecosystem with universal access to care and free choice of center, the opposite occurs: the outsourced hospitals attend more complex patients. Interestingly, no differences were observed in inpatient complication rates during the study period, despite higher patient complexity in study hospitals, suggesting that care delivery and management practices in value-based hospitals can improve patient outcomes despite higher levels of comorbidity [40,41,42].

Efficiency gains were also observed, particularly in reduced average inpatient length of stay and lower-than-expected case-mix-adjusted lengths of stay. These results align with the goals of value-based healthcare to optimize resource utilization without compromising outcomes [7,8]. Shorter hospital stays are associated with lower costs, decreased risk of complications, and improved patient flow, which are critical for regional health systems under increasing demand pressures [43,44,45]. The implementation of outpatient and emergency department initiatives to reduce length of stay and unnecessary hospital visits in study hospitals has also proven effective in improving patient and clinician experience [46,47,48,49].

Patient satisfaction outcomes were consistently superior in study hospitals. Higher satisfaction and recommendation scores, combined with increased patient transfers toward these hospitals under the Free Choice of Care Mandate, suggest that patients perceive tangible benefits in receiving care within the value-based network, in line with other studies [17,47,50]. Patient choice, in turn, may reinforce quality improvements by incentivizing providers to deliver care that aligns with patient expectations and experiences.

These findings build on prior studies suggesting that value-based models can enhance healthcare delivery in tertiary hospital settings. Our results extend this evidence to low- and intermediate-complexity hospitals, which play a central role in regional healthcare systems as entry points for patients. A previous work in Spain highlighted improved safety, efficiency, and satisfaction in high-complexity hospitals managed under value-based arrangements [17]. The present study demonstrates that similar benefits may be realized at lower levels of hospital complexity, supporting the scalability of this model across different tiers of healthcare delivery.

The coexistence of public and privately managed hospitals within the Madrid regional system offers a unique opportunity to evaluate hybrid models of healthcare delivery in Europe. Our findings suggest that value-based outsourcing may enhance system-wide performance, particularly in regions where hospital complexity stratification allows for meaningful comparison. Importantly, because universal coverage and zero out-of-pocket costs are guaranteed across both models, differences in outcomes cannot be explained by financial barriers to care, but rather by differences in organizational structure and management [34]. From a policy perspective, these results provide initial support for considering value-based outsourcing as a mechanism to improve efficiency and patient outcomes in lower-complexity hospital settings. However, successful implementation likely depends on ensuring robust governance, transparency in performance monitoring, and strong alignment of incentives between public authorities and private providers.

This study has several strengths. It included all low- and intermediate-complexity hospitals within the Madrid region, minimizing selection bias, and relied exclusively on standardized audit data from the regional health authority, ensuring comparability across hospitals. The use of adjusted indicators, such as case-mix standardized mortality and length of stay, allowed for fairer benchmarking across institutions with different patient profiles. Nonetheless, some limitations should be acknowledged. First, the cross-sectional design precludes causal inference, and unmeasured confounding factors (such as differences in staffing, infrastructure, or referral patterns) may have influenced the results. Secondly, and most importantly, the audit data did not directly report the total number of patients included in the calculation for hospital-acquired infection, and so the researchers estimated this total based on the annual number of inpatients during the study period. While we acknowledge that this may have resulted in slight differences in our results, we believe that the adoption of a common methodology following European standards in order to calculate the prevalence of hospital-acquired infection ensures the validity of our analysis. Thirdly, while patient satisfaction surveys provide valuable insights into patient experience, they may be subject to response bias, and the excellent level of patient satisfaction for hospitals in the Madrid area could limit the sensitivity of our analysis. Finally, these findings may not be generalizable outside the Madrid context, where hospital complexity stratification and the Free Choice of Care Mandate create unique conditions for comparison. Longitudinal studies and qualitative assessments would be valuable to better understand the mechanisms driving observed differences.

The results point to the importance of a data-driven approach to outsourcing as part of a sustainable solution to the current healthcare quandary. In our study, the available data demonstrates that value-based outsourcing may be a solution to increase efficiency while maintaining high patient safety, quality of care, and patient satisfaction. Implications for further research include qualitative studies to investigate perspectives of healthcare managers and clinical leaders participating in value-based outsourcing, as well as prospective studies in different healthcare systems to validate the generalizability of our findings.

## 5. Conclusions

In summary, value-based outsourcing of low- and intermediate-complexity hospitals in Madrid was associated with improved efficiency defined as lower length of hospital stay and case-mix-adjusted length of stay, similar rates of inpatient complications, lower infection and cesarean section rates, and higher patient satisfaction scores and inward transfers, despite higher case-mix complexity compared to publicly managed and non-value based outsourced hospitals. These findings support the potential of value-based healthcare networks to enhance performance beyond tertiary hospitals, offering lessons for healthcare systems seeking to optimize resource use while maintaining universal access and high standards of care. Future research should explore the long-term sustainability of these outcomes and their applicability in other healthcare settings.

## Figures and Tables

**Table 1:** Differences between hospital levels of complexity from the Regional Madrid Healthcare System.

Characteristic	Level 1 Hospitals (Low Complexity)	Level 2 Hospitals (Intermediate Complexity)	Level 3 Hospitals (High Complexity)
Hospital Type	Regional or local area hospitals.	General intermediate-sized hospitals.	Referral and tertiary-level hospitals, with large size and high activity.
Services and Specialties	Offer basic medical and surgical services. They usually have core specialties such as internal medicine, pediatrics, obstetrics and gynecology, and general surgery.	Have a broader range of specialties and subspecialties. They include intensive care units (ICUs) and a greater provision of diagnostic and therapeutic technology.	Have all medical-surgical specialties, including the most complex and subspecialties. They are referral centers for rare diseases, transplants, advanced oncology, and high-complexity surgery.
Technology and Equipment	Basic technological equipment.	Advanced technology, but without the most specialized units. For example, they may have an MRI or CT scanner but not PET or cyclotron units.	Have the highest technology, including very advanced diagnostic imaging equipment (PET-CT), and robotic surgery technology.
Teaching and Research Capacity	Little to no teaching capacity. They are not usually university hospitals or research centers.	Have greater teaching activity and often have agreements with universities for the training of medical residents (MIR).	They are university hospitals and leading research centers. They have a large number of residents and are highly involved in the training of future specialists.

**Table 2:** Operational definitions of healthcare outcomes and other indicators included in the study.

Quality of Care and Patient Safety Outcomes	
Medical and surgical inpatient complications	Number of discharged patients with one or more ICD-10 coded complications divided by the total number of patients discharged during the year, multiplied by 100
Hospital-acquired infections	Number of patients with a hospital-acquired infection during a predetermined period divided by the total number of inpatients during that period, multiplied by 100
Low-risk cesarean sections	Number of cesarean sections performed in women with low-risk pregnancies (absence of abnormal presentation, preterm birth, stillbirth, and multiple pregnancies), divided by total number of births during the year, and multiplied by 100
**Efficiency Outcomes**	
Length of hospital stay	Average length of hospital inpatient stay in days during the year
Case-mix-adjusted average inpatient length of stay	Average length of hospital inpatient stay (in days) divided by the reference standard (the estimated length of stay in days, adjusted by case mix and based on the RMHS overall mean) during the year
**Patient Satisfaction Outcomes**	
Patient satisfaction scores	Results of patient satisfaction surveys where 0 is the worst possible experience and 100 is the best possible experience
Inward transfers	Number of patients opting to transfer to a hospital outside of their catchment area per year

**Table 3:** Care episodes and indicators included in the analysis.

Variable	Study Group	Control Group	*p*-Value
Total care episodes	1,743,090	5,941,372	N/A
Total inpatient hospital stays	46,632	167,247	N/A
Total births	3473	13,088	N/A
Inpatient medical and surgical complications	1192 (2.58%)	4320 (2.56%)	0.007
Hospital-acquired infections *	1620 (3.47%)	9130 (5.46%)	<0.001
Low-risk cesarean sections	559 (16.10%)	2529 (19.32%)	<0.001

* Number of total hospital-acquired infections during the study period was estimated by multiplying the reported percentage of hospital-acquired infections in point-prevalence surveys by the total number of inpatient hospital stays during the study period.

## Data Availability

All underlying data are available at https://www.comunidad.madrid/servicios/salud/observatorio-resultados-servicio-madrileno-salud (accessed on 26 March 2024). The minimal dataset used for analysis is also available as Supplementary Materials.

## Supplementary Materials

The following supporting information can be downloaded at: https://www.mdpi.com/article/10.3390/healthcare14060731/s1.
